# Comparison of telomere length and association with progenitor cell markers in lacrimal gland between Sjögren syndrome and non-Sjögren syndrome dry eye patients

**Published:** 2011-05-27

**Authors:** Motoko Kawashima, Tetsuya Kawakita, Yoshiko Maida, Mizuka Kamoi, Yoko Ogawa, Shigeto Shimmura, Kenkichi Masutomi, Kazuo Tsubota

**Affiliations:** 1 Department of Ophthalmology, Keio University School of Medicine, Tokyo, Japan; 2 Cancer Stem Cell Project, National Cancer Center Research Institute, Tokyo, Japan

## Abstract

**Purpose:**

Indicators of aging such as disruption of telomeric function due to shortening may be more frequent in dysfunctional lacrimal gland. The aims of this study were to 1) determine the viability of quantitative fluorescence in situ hybridization of telomeres (telo-FISH) for the assessment of telomere length in lacrimal gland in Sjögren and non- Sjögren syndrome patients; and 2) investigate the relationship between progenitor cell markers and telomere length in both groups.

**Methods:**

Quantitative fluorescence in situ hybridization with a peptide nucleic acid probe complementary to the telomere repeat sequence was performed on frozen sections from human lacrimal gland tissues. The mean fluorescence intensity of telomere spots was automatically quantified by image analysis as relative telomere length in lacrimal gland epithelial cells. Immunostaining for p63, nucleostemin, ATP-binding cassette, sub-family G, member 2 (ABCG2), and nestin was also performed.

**Results:**

Telomere intensity in the Sjögren syndrome group (6,785.0±455) was significantly lower than that in the non-Sjögren syndrome group (7,494.7±477; p=0.02). Among the samples from the non-Sjögren syndrome group, immunostaining revealed that p63 was expressed in 1–3 acinar cells in each acinar unit and continuously in the basal layer of duct cells. In contrast, in the Sjögren syndrome group, p63 and nucleostemin showed a lower level of expression. ABCG2 was expressed in acinar cells in both sjogren and non-Sjogren syndrome.

**Conclusions:**

The results of this study indicate that 1) telo-FISH is a viable method of assessing telomere length in lacrimal gland, and 2) telomere length in Sjögren syndrome is shorter and associated with lower levels of expression of p63 and nucleostemin than in non-Sjögren syndrome.

## Introduction

Telomeres are specialized DNA sequences located at the ends of chromosomes which shorten with each successive round of cell division. Accumulating evidence indicates that telomere length in human somatic cells shortens with chronological aging [1,2]. The maximum number of possible cell divisions in a given cell population is fixed. It has been suggested that this replicative life span, also known as the “Hayflick limit,” is determined by the telomere having a “critical” length [3]. In human, telomere length has been measured extensively in leukocytes in relation to chronological aging [4-6]. Recently, telomere length was reported to decline with age in mature endothelial cells and to contribute to endothelial dysfunction and atherogenesis [7-9].

Alteration in telomere length may play a role in the development of several diseases in human, including cancer and benign inflammatory diseases such as idiopathic pulmonary fibrosis and type 2 diabetes [10-15]. On the other hand, some studies have indicated that pathological stresses themselves may affect telomere shortening, with inflammation, for example, reported as one possible cause [10-12,14], perhaps due to the concomitant increase in turnover of cells. Sjögren syndrome is a chronic inflammatory disease affecting the lacrimal glands [16,17]. We hypothesized that telomere length shortening in lacrimal gland was related to inflammation of lacrimal gland.

Flores et al. [18] reported that telomeres shorten with age in mouse stem cells from various tissues, suggesting that telomere loss contributes to stem cell dysfunction with aging. In addition, they also reported that the longest telomeres were a general feature of the adult stem cell compartment. Although no reports have demonstrated the presence of stem cells in lacrimal gland, tissue-committed progenitor cells are believed to be present. A recent report showed that injured lacrimal gland can undergo repair after acinar cells are lost through apoptosis or autophagy, which is followed by an increase in the number of stem/progenitor cells, stimulation of proliferation and upregulation of the bone morphogenetic protein 3 (BMP7) pathway [19]. We hypothesized that progenitor cell markers reported in the corneal and conjunctival epithelia, which are of the same origin as the lacrimal gland during development, were related to telomere shortening. Therefore we selected p63, nucleostemin, ATP-binding cassette, sub-family G, member 2 (ABCG2), and nestin as progenitor cell markers for this study. p63 has been recognized as markers for epithelial cells which have potential to proliferate and stratified [20]. Nucleostemin has been reported to be related to small cell size and similar expression pattern as p63 in corneal epithelium [21,22]. ABCG2 has been identified as a molecular determinant for bone marrow stem cells and proposed as a universal marker for stem cells including corneal limbal epithelial stem cells [23,24]. Nestin has been used as progenitor marker in the study of lacrimal gland tissue repair after injury [19,25].

To the authors’ knowledge, no studies have been published on telomere shortening in lacrimal gland. Therefore, the aims of this study were to 1) determine the viability of quantitative fluorescence in situ hybridization (FISH) of telomeres (telo-FISH) in the assessment of telomere length in lacrimal gland in Sjögren and non-Sjögren syndrome patients; and 2) investigate the relationship between progenitor cell markers and telomere length in both groups.

## Methods

### Tissue samples

Human tissue samples were obtained with written informed consent from patients treated at the Department of Ophthalmology, Keio University Hospital, Tokyo, Japan. These were in accordance with the principles expressed in the Declaration of Helsinki. The approval of the Keio University Ethics Committee was obtained for the use of human materials for this research. Lacrimal gland biopsy specimens were collected from 11 patients with dry eye to determine the presence or absence of Sjögren syndrome. Sjögren syndrome diagnosis was based on the revised American-European consensus criteria [26,27]. For the lacrimal gland study, normal lacrimal gland controls were not available for ethical reasons. Thus, we used lacrimal glands biopsy samples that were obtained for diagnostic purposes.

All tissue samples were routinely embedded in Optimal Cutting Temperature (OCT) compound and stored at −80 °C. Some of the samples were processed for electron microscopy. Frozen blocks were sectioned to a thickness of 5 μm and used for telo-FISH, immunohistochemical analyses and hematoxylin and eosin (H&E) staining.

### Telomere-FISH analysis

Telo-FISH was performed using a 5′-Cy3-labeled, telomere-specific peptide nucleic acid (PNA) probe (5′-CCC TAA CCC TAA CCC TAA-3′; Fasmac, Kanagawa, Japan) as described previously with some modifications [28]. Sections were fixed with 4% paraformaldehyde (PFA). Slides were incubated with 10 mM sodium citrate at 80 °C for 30 min. After washing with 1× PBS, slides were dehydrated using an ethanol series (25%, 50%, and 100%) and air-dried. Twenty microliters Cy3-labeled telomere-specific PNA probe (2 μg/ml PNA in 70% formamide buffer with blocking reagent and 10 mM MgSO_4_) was added to the sample and denaturation then performed by incubation for 15 min at 95 °C. Slides were incubated overnight at 37 °C for hybridization. Slides were then washed in 70% formamide buffer 4× for 15 min each time, followed by washing in PBS–0.025% Tween-20 four times for 5 min each. Slides were then dehydrated using an ethanol series and air-dried. The nuclei were stained with DAPI (Molecular Probes, Eugene, OR) for 30 min. The slides were then washed in PBS for 1 min, briefly dehydrated using the ethanol series and air-dried. The slides were then mounted with Vectashield (Vector Laboratories, Burlingame, CA).

### Image capturing and analysis

Tissue structure was identified on H&E-stained adjacent tissue sections before fluorescent microscopy. For telo-FISH, fluorescence images of DAPI, and Cy3 were recorded with an Olympus IX-81 inverted fluorescence microscope with spinning disk confocal unit (IX81-DSU; Olympus, Tokyo, Japan) equipped with a cooled CCD ORCA-AG camera (HAMAMATSU, Shizuoka, Japan) using a UplanSApo 100× /NA.1.40 (Olympus) oil immersion objective lens. All quantitative image analysis were analyzed in MetaMorph 7.6.3.0 software (MDS Analytical Technologies, Sunnyvale,CA). Exposure times were optimized with respect to the intensities of telomere and nuclear signals to prevent overexposure/saturation and kept constant for all slides to ensure consistency in intensity measurement. To avoid differences due to variation in section thickness, we used slices of the same thickness (5 μm) in all tissues analyzed.

The DAPI image was used to define the nuclear area and the Cy3 image was used to quantify telomere fluorescence. The nuclei of lacrimal gland cells were outlined based on the DAPI signal, followed by outlining of the telomere signals in individual nuclei. The intensities of all outlined pixels of both telomere signals and DAPI were summed respectively on a per-cell basis and tabulated. Inflammatory cells were excluded from the analysis. Telomere signals were standardized with DAPI signals and telomere intensity was calculated for each nucleus as follows: telomere intensity (TI)=(sum of all telomere signal intensities)/(intensity of DAPI signal). Calculation at each step in the analysis was performed automatically. Ten fields were examined for each sample, and average telomere intensity in each sample calculated.

### Statistical analysis

We used the Student *t* -test to analyze the results of the clinical findings and the Mann–Whitney *U*-test to analyze the results of telo-FISH. A probability value of p<0.05 was considered to indicate statistical significance.

### Immunohistochemistry

Lacrimal gland tissue slides were fixed with 2% paraformaldehyde (PFA; Wako, Osaka, Japan) for immunostaining for p63 and nucleostemin. After background staining was blocked with 10% normal donkey serum, samples were treated with the following monoclonal primary antibodies: anti-p63 (4A4; Calbiochem, A Brand of EMD Biosciences, Inc., San Diego, CA), anti-nucleostemin (R&D Systems, Minneapolis, MN), anti-ABCG2 (Chemicon, Millipore, Billerica, MA), and anti-nestin (Santa Cruz Biotechnology, Santa Cruz, CA). Samples were then treated with Cy3 (Jackson ImmunoResearch, West Grove, PA)-conjugated secondary antibodies. Nuclei were counterstained with 4',6'-diamino-2-phenylindole (1 mg/ml, DAPI; Dojindo Laboratories, Tokyo, Japan).

### Transmission electron microscopy

To investigate the structural change of cells in both groups, we performed transmission electron microscopy analysis. A portion of lacrimal gland tissue was immediately fixed with 2.5% glutaraldehyde and subjected to electron microscopic examination as described previously [29]. One-micrometer-thick sections were stained with methylene blue and portions exhibiting lacrimal gland structure thin-sectioned and examined with an electron microscope (1200 EXII; JEOL, Tokyo, Japan).

## Results

### Demographic data

Profiles of the patients are shown in Table 1. A diagnosis of Sjögren syndrome was made in 4 of 11 patients and non-Sjögren syndrome in 7 patients. Patients ranged in age from 24 to 74 years (mean±SD age, 55.7±14.3 years). No significant difference was found in age of Sjögren (48.3±17.6 years) and non-Sjögren syndrome (60±11.1 years) patients. Sjögren syndrome had significantly higher Rose Bengal score than non-Sjögren syndrome (p<0.001), which was the only significant difference among the clinical parameters between the two groups.

**Table 1:** Patient's demographic data and telomere intensity.

**Number**	**Diagnosis**	**Age**	**Fluorescein score**	**Rose bengal score**	**Schirmer value**	**Schirmer value with nasal stimulation**
1	SS	24	6	8	2	1
2	SS	49	1	7	5	NA
3	SS	54	2	NA	2	NA
4	SS	66	9	9	5	4
**mean±SD**	** **	**48.3±17.6**	**4.5±3.6**	**8.0±1.0**	**3.5±1.7**	**2.5±2.1**
5	Non-SS	42	1	4	2	35
6	Non-SS	54	6	2	8	NA
7	Non-SS	54	0	2	2	16
8	Non-SS	59	3	0	5	7
9	Non-SS	66	0	0	4	24
10	Non-SS	71	2	NA	4	2
11	Non-SS	74	0	2	4	4
**mean±SD**	** **	**60.0±11.1**	**1.7±2.2**	**1.7±1.5**	**4.1±2.0**	**14.7±12.9**

SS, Sjögren syndrome; Non-SS, Non-Sjögren syndrome; NA, Not applicable; SD, standard deviation.

### Telomere shortening in Sjögren syndrome

**Figure 1:**
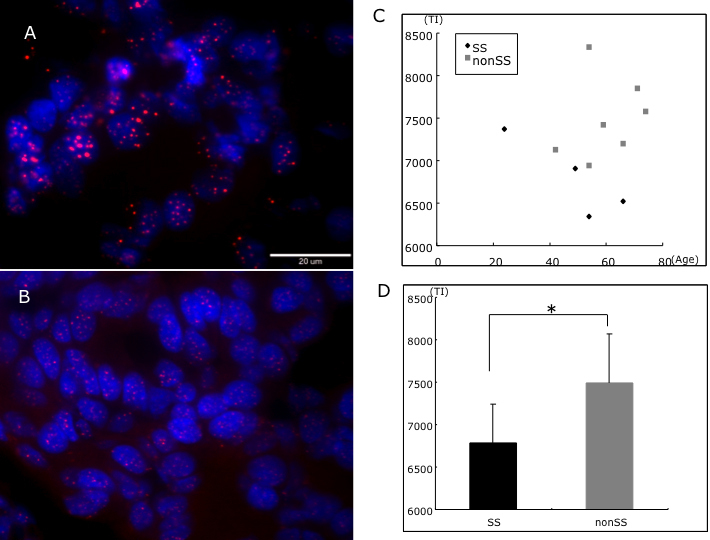
Telomere shortening in Sjögren syndrome. Representative telo-FISH photograph of non-Sjögren syndrome (**A**) and Sjögren syndrome (**B**) lacrimal glands. Scatter plot of age and telomere intensity (TI; **C**). Comparison of average telomere intensity in Sjögren and non-Sjögren syndrome groups demonstrated that telomeres were significantly shorter in Sjögren syndrome group (**D**; p=0.02).

Large numbers of inflammatory cells invade lacrimal gland tissue in Sjögren syndrome. Therefore, we selected only locations where acinar unit structure was well preserved for telo-FISH. In this study, telo-FISH was successfully performed on fixed frozen tissue sections. Representative photos are shown in Figure 1A,B. High levels of Cy3 expression were observed in the nuclei in lacrimal gland in the non-Sjögren syndrome group, whereas expression was weak in the Sjögren syndrome group (Figure 1A,B). Telomere intensity in lacrimal gland epithelial cells in the Sjögren syndrome group (6,785.0±455) was significantly lower than that in the non-Sjögren syndrome group (7,494.7±477; p=0.02, Figure 1C,D).

### Progenitor cell markers showed relationship with telomere shortening

**Figure 2:**
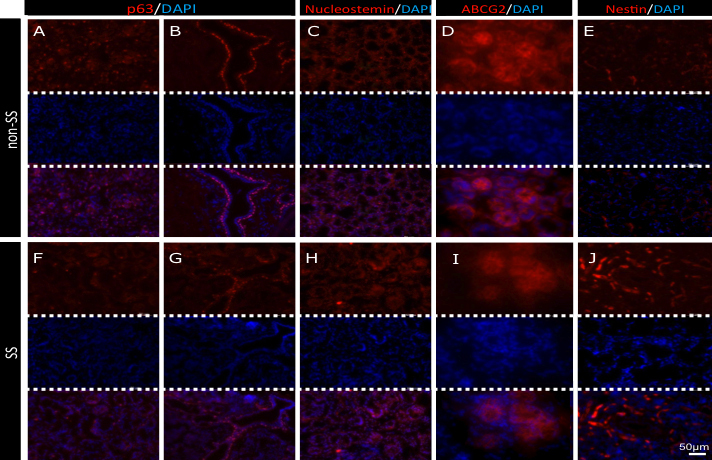
Immunostaining for progenitor markers. In non-Sjögren syndrome, p63 (red) was expressed in 2–4 cells in each acinar unit (**A**) and all ductal basal cells (**B**; case 7). In Sjögren syndrome, p63 was weakly expressed with irregular pattern. (case 2; **F**, **G**). Nucleostemin was expressed with a similar pattern in non-Sjögren syndrome (case 7; **C**) and Sjögren syndrome (case 2; **H**). Nuclei were counterstained with DAPI (blue). ABCG2 (red) was expressed in intercellular junction and cytoplasm in acinar unit (**F**, **I**), and weaker in Sjögren syndrome. Nestin was expressed strongly in some location in Sjögren syndrome (**E**, **J**). Scale bars indicate 50 μm (**A**-**C**, **E**-**H**, **J**) and 20 μm (**D**, **I**), SS=Sjögren syndrome, non-SS=non Sjögren syndrome.

To investigate the relationship between telomere length and progenitor cell markers, we performed immunostaining for p63 and nucleostemin. In the non-Sjögren syndrome group, p63 was expressed in 2–4 acinar cells in each acinar unit (Figure 2A) and in the basal layer of duct basal cells continuously (Figure 2B). In contrast, p63 showed a lower level of expression and was only sparsely expressed in the basal layer of duct cells in the Sjögren syndrome group (Figure 2F,G). Nucleostemin showed a similar pattern of expression as p63, being higher and more regularly expressed in the non-Sjögren syndrome group than in the Sjögren syndrome group (Figure 2C,H). ABCG2 was expressed in intercellular junction and cytoplasm of most acinar unit regularly in the non-Sjögren syndrome group (Figure 2D). Whereas acinar unit was decreased in the Sjögren syndrome group, and acinar unit with ABCG2 expression was also decreased (Figure 2I). Nestin was expressed outside the acinar unit in both group, Elongated cells with nestin expression were observed more frequently in the SS group, compared with the non-SS group. Those nestin-positive cells were not observed uniformly in all locations, but formed cell clusters at tissue damaged areas in the SS group (Figure 2F,J). Telomere length was shorter and expression of progenitor markers weaker or non-existent in the Sjögren syndrome group.

### Electron microscopy findings

**Figure 3:**
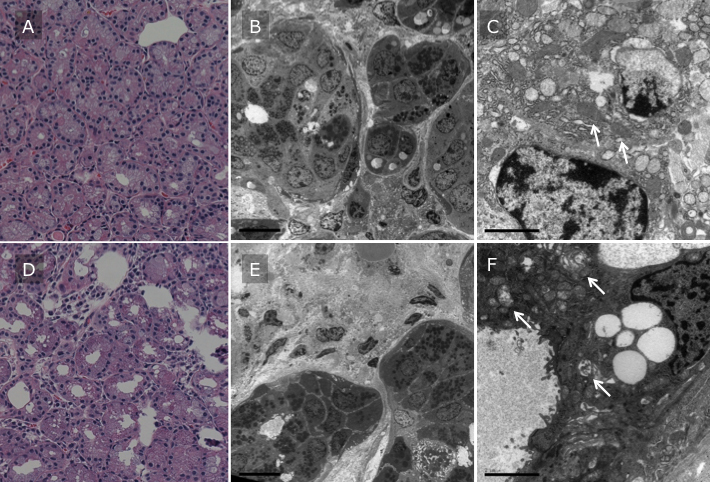
Electron microscopy of lacrimal gland. Representative H&E staining and electron microscopy photographs of non-Sjögren syndrome (case 9; **A**, **B**, **C**) and Sjögren syndrome patient (case 4; **D**, **E**, **F**) were shown. Scale bar indicated 5 μm (**B** and **E**) and 2 μm (**C** and **F**). Structure of lacrimal acinar unit was compact and uniform in non-Sjögren syndrome patients (**A** and **B**), but mild acinar atrophy and fibrosis were observed more frequently in Sjögren syndrome patients (**D** and **E**). High magnification revealed that structure of mitochondrial cristae (arrows) was severely damaged and swollen in Sjögren syndrome patient (**F**) compared to that in non-Sjögren syndrome patient (**C**).

To determine ultrastructural changes in lacrimal gland, we analyzed samples using electron microscopy. Electron microscopy revealed that the structure of each lacrimal acinar unit was compact and uniform in the non-Sjögren syndrome group. Myoepithelial cells were smooth in shape (Figure 3B). Mild acinar atrophy and fibrosis were observed more frequently in the Sjögren syndrome group (Figure 3E). Infiltration of inflammatory cells was observed in both groups, being particularly marked in the Sjögren syndrome group. High magnification revealed that the structure of mitochondrial cristae was severely damaged and swollen in the Sjögren syndrome group (Figure 3F) compared to that in the non-Sjögren syndrome group (Figure 3C), indicating that mitochondrial damage may be related to Sjögren syndrome.

## Discussion

In this study, we successfully measured telomere intensity in lacrimal gland epithelial cells by telo-FISH and investigated relative telomere length in each cell. We believe that this is the first report of a telomere length analysis in lacrimal gland.

The results showed that the telomeres in lacrimal gland cells in the Sjögren syndrome group were significantly shorter than those in the non-Sjögren syndrome group (p=0.02). Patchy invasion by inflammatory cells and the destruction of lacrimal gland structure were observed frequently in the Sjögren syndrome group. It should be noted that these results were obtained even though we selected only those areas in which acinar unit structure was preserved for telo-FISH. Furthermore, even though the clinical findings were similar between the two groups, telomere length showed a significant difference.

These results suggest that telomere length is related to severe dry eye diseases where normal lacrimal gland function has been disrupted by chronic inflammation. Recently, it has been reported that renal failure shortens cardiac telomeres, and that short telomeres are a risk factor for idiopathic pulmonary fibrosis [10,11]. The present results are consistent with these earlier reports indicating a strong association between organ dysfunction and telomere shortening, suggesting telomere shortening as a risk factor for lacrimal gland dysfunction as well. Telomere shortening has been reported in several inflammatory diseases such as vascular disease, type 2 diabetes, Fanconi anemia, and ataxia teleangiectasia [12-15]. Lacrimal gland epithelial cell turnover was not clearly defined until recently, however, so further study is necessary to investigate the relationship between telomere length and inflammation in this tissue.

Progenitor cell marker expression (p63, nucleostemin, and ABCG2) was weaker in the Sjögren syndrome group than in non-Sjögren syndrome group. p63 is often used as a progenitor cell marker for keratinocytes and corneal epithelial cells with high proliferative potential [20]. Nucleostemin has been reported in proliferating cells in various tissues, including bone marrow, and may be used as a progenitor marker for stratified epithelial cells [21,22]. ABCG2 and nestin have been recognized as one of progenitor cell markers in adult tissue. Surprisingly, nestin-positve cells were observed more frequently in the Sjögren syndrome, which maybe partially explained that nestin expressed only in repairing/regeneration location, but not in quiescent cells [19,25]. These results suggest that telomere length shortening in lacrimal progenitor cells indicates the pathophysiological conditions necessary for development of Sjögren syndrome. Although most tissues are known to have their own tissue-specific stem cells, the existence of stem cells in lacrimal gland has yet to be proven. The results of telo-FISH may indicate the existence of progenitor cells in lacrimal gland [30]. Further investigation is needed to characterize the progenitor cells and their homeostasis in lacrimal gland.

Electron microscopy revealed that the structure of mitochondrial cristae was severely damaged in the Sjögren syndrome group (Figure 3F) compared to in the non-Sjögren syndrome group (Figure 3C). There were some reports about the associations among telomere length, mitochondrial function and oxidative stress [31-36]. Mitochondria are the most important source of reactive oxygen species in cells under physiologic conditions, and premature senescent cells sorted from young cultures displayed mitochondrial dysfunction, increased oxidative stress and short telomeres [31]. Another report showed that improvement in mitochondrial function results in less telomeric damage and slower telomere shortening, while telomere-dependent growth arrest is associated with increased mitochondrial dysfunction [32]. Furthermore, telomere-shortening rate and cell replicative life spans can be greatly modified by DNA-damaging oxidative stress [5], which has been shown to accelerate telomere shortening during DNA replication [37]. The mitochondrial structural changes observed in this study may contribute to the increase in oxidative stress induced by Sjögren syndrome. However the relationship between mitochondrial damage and telomere shortening was not clear in this study, and further study was necessary to clarify the molecular mechanism. The results of this study indicate that 1) telo-FISH is a viable method of assessing telomere length in lacrimal gland; and 2) telomere length in Sjögren syndrome is shorter than in non-Sjögren syndrome, possibly due to acceleration of the cell cycle to maintain lacrimal gland cell homeostasis, and associated with lower levels of expression of p63 and nucleostemin.

Taken together, this suggests that dysfunction in lacrimal gland may be related to epithelial cell telomere shortening. Further study is needed, however, to clarify the underlying molecular mechanism.
